# The Influence of Art in the School-Room

**Published:** 1913-06

**Authors:** John B. Sherwood

**Affiliations:** 1505 Monroe Street, Chicago


### The Influence of Art in the School-Room.

The value of art in its influence upon the mind and character of the young is growing to be better understood by everyone interested in our modern education. Surrounded by beauty the child's expression of life becomes more beautiful in response to an environment which refines and purifies, and which stimulates his thought to higher ideals.

Pictures, and other works of Art, on the school-room wall, exert a powerful influence on the mind of the child. In learning to love the beautiful he learns also to love the good and the true.

This influence has a direct effect upon speech and manners; both boys and girls "grow in grace" when surrounded by beauty which appeals to their higher nature.

In many of our public schools a happier atmosphere is apparent since school-rooms are being changed from the bare, desolate, uninteresting places we can remember, to rooms which are restful and harmonious in their color, and in their art decoration an inspiration and delight to every child.

It is important to choose for the school-room those subjects in Art which will best develop the nobler instincts. Many of the city children have never seen a meadow or the country in the spring time. They know nothing of the sweet delights of nature. In those bred to city life how shall we keep alive this love of nature—one of the purest joys of life? By placing before their eyes the pictures of nature's sweetest haunts which great artists have reproduced for us.

The city child can learn to love beautiful nature from beautiful pictures. Robert Browning knew this when he said:

> "We're made so that we love,
> First when we see them painted, things we have passed
> Perhaps a hundred times nor cared to see."

The country child, though surrounded by the beauty of woods and fields, yet lacks the refining influence which Art gives in its interpretation of Nature, and so should have placed in the school-room casts of noble sculpture, and reproductions of the greatest achievements of mankind in architecture and painting.

Every teacher feels the value of artistic surroundings in the school-room. The refining, uplifting influence of classic form, harmonious color and storied suggestion of beautiful lives with which a school-room may be filled, cannot but be helpful to the teacher and educative to the child.

A well known artist and writer—William Ordway Partridge, of Boston—speaks in no uncertain way of this very subject. He says in a late book—*Art for America*: "We should have no blank walls in our school-rooms. It is just as important to hang reproductions of great paintings and frescoes upon the walls as it is to place books under the children's eyes. We cannot have a great art until art education is more generally taught in the public schools. Let the starved natures of the children in our great city schools learn to know that outside those brick walls there is a world of surpassing loveliness. This they may learn through seeing beautiful pictures and beautiful statuary."

Our Commissioner of Education says in the *Educational Review*: "The greatest works of art should become the ones most familiar to the people. Care should be taken, therefore, to select for a school only these great works, to lead the pupil into an understanding of the underlying beauty which is in the world, but is discovered only by eyes familiar with and trained to see and know its highest form."

One of the most promising signs of the times is this new factor in the "higher education." The curriculum of study in our public schools cannot be better strengthened than by making the history of great art and something of its technique a part of the regular study course.

This is being most successfully done in some of our cities. The young people are studying eagerly the greatest architecture, sculpture and painting of the world, with the aid of large photographs pinned upon the walls to illustrate each phase of art. In Indianapolis, Ind., it is said that the conditions are more nearly like those of Florence in the beginning of that great "Renaissance" of art in the fourteenth century; artists and citizens, high and low, rich and poor, contribute to the upbuilding of art by their universal interest in art matters—by the support of their artists and the decoration of their homes and schools.

The influence of such an environment and the value of such training upon young eyes and hearts to see and know and choose the beautiful, cannot but make a difference in the character of those who will become the workers of the coming century.

MRS. JOHN B. SHERWOOD.

1505 Monroe Street, Chicago. Jan. 20, 1900.

